# Towards an improved internet of things sensors data quality for a smart aquaponics system yield prediction

**DOI:** 10.1016/j.mex.2023.102436

**Published:** 2023-10-11

**Authors:** A.H. Eneh, C.N. Udanor, N.I. Ossai, S.O. Aneke, P.O. Ugwoke, A.A. Obayi, C.H. Ugwuishiwu, G.E. Okereke

**Affiliations:** a Department of Computer Science, University of Nigeria, Nigeria; b Department of Zoology & Environmental Biology, University of Nigeria, Nigeria; c Digital Bridge Institute, Nigeria Communications Commission, Abuja, Nigeria

**Keywords:** Internet of things, Cloud computing, Sensors, Aquaculture, Catfish, Machine learning, Improved Smart Aquaponics Dataset Collection Techniques

## Abstract

The mobile aquaponics system is a sustainable integrated aquaculture-crop production system in which wastewater from fish ponds are utilized in crop production, filtered, and returned for aquaculture uses. This process ensures the optimization of water and nutrients as well as the simultaneous production of fish and crops in portable homestead models. The Lack of datasets and documentations on monitoring growth parameters in Sub-Saharan Africa hamper the effective management and prediction of yields. Water quality impacts the fish growth rate, feed consumption, and general well-being irrespective of the system. This research presents an improvement on the IoT water quality sensor system earlier developed in a previous study in carried out in conjunction with two local catfish farmers. The improved system produced datasets that when trained using several machine learning algorithms achieved a test RMSE score of 0.6140 against 1.0128 from the old system for fish length prediction using Decision Tree Regressor. Further testing with the XGBoost Regressor achieved a test RMSE score of 7.0192 for fish weight prediction from the initial IoT dataset and 0.7793 from the improved IoT dataset. Both systems achieved a prediction accuracy of 99%. These evaluations clearly show that the improved system outperformed the initial one.

• The discovery and use of improved IoT pond water quality sensors.
• Development of machine learning models to evaluate the methods.
• Testing of the datasets from the two methods using the machine learning models.

## Graphical abstract

**Figure:**
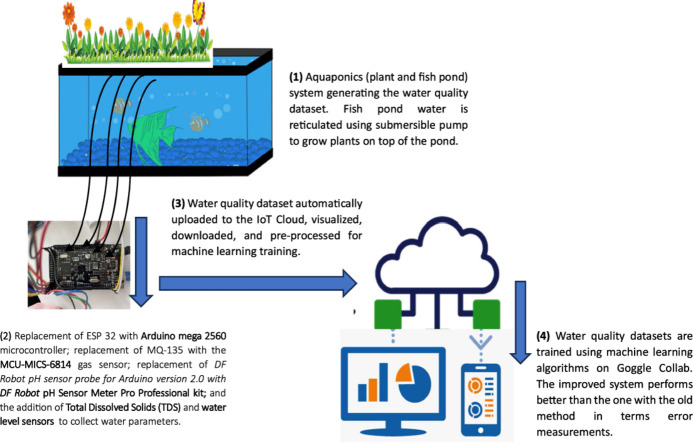

Specifications table

**Table:** 

Subject area:	Computer Science
More specific subject area:	Application of Computer Science (IoT Sensors and Machine learning) to Aquaculture
Name of your method:	Improved Smart Aquaponics Dataset Collection Techniques
Name and reference of original method:	A Pilot Implementation of a Remote IoT Sensors for Aquaponics System Datasets Acquisition **C.N. Udanor**, N.I. Ossai, B.O. Ogbuokiri, O.E. Nweke, P.O. Ugwoke, U.K. Ome (2021). A Pilot Implementation of a Remote IoT Sensors for Aquaponics System Datasets Acquisition, The Journal of Computer Science and it's Applications (JCSA), Vol. 28, Iss. 2. https://dx.doi.org/10.4314/jcsia.v28i2.1
Resource availability:	Dataset: dataset at: https://www.kaggle.com/datasets/ogbuokiriblessing/sensor-based-aquaponics-fish-pond-datasets Initial method: https://www.sciencedirect.com/science/article/pii/S2352340922005972

## Method details

Several previous IoT sensor-based systems for monitoring water quality in aquaculture employed a combination of sensors like temperature, pH value, dissolved oxygen, and water level, among others [4]. Chen, et. al. [5] in Taiwan used the Arduino Mega 2560 microcontroller and transmitted the water quality dataset over a LoRaWAN network. Danh et al. [6] in Vietnam added salinity and oxidation-reduction sensors and used the Thingspeak cloud storage, providing a mobile user interface for farmers. Nocheski and Naumoski [7] added a light intensity sensor in addition to the other water quality sensors listed above. Saha et al. [8] used Raspberry Pi as the microcontroller to implement pond water quality monitoring with a smartphone camera and an Android application. Kim et al. [9] in addition implemented a closed-loop water flow control using the Message Queue Telemetry Transport (MQTT) communication protocol. Taher, et al. [10] helps farmers in Bangladesh to monitor the health of their fish using a combination of dissolved oxygen, pH, and ammonia sensors. In helping farmers monitor their fish farms [11] introduce a QR code tag of an aquatic product to track and view historical data. Ramya et al. [12] employed IoT to remotely monitor the quantity of food items in the pond water, as well as implementing an automatic feeding system.

Other digital technologies employed to fish farm data collection and analysis include AI, big data analytics, and blockchain [13], web-based applications with real-time sensor data visualization, alert and remote-control water pump systems [14]. Machine learning algorithms like logistic regression are also used to predict fish disease by analyzing the IoT water quality data collected in Bangladesh [15].

We observed that while a handful of work has been done on IoT-based aquaculture systems mostly in Asia, not much work has been done in the Sub-Saharan African region, and neither is much work done on aquaponics IoT combination. Yet also there is little or no public water quality dataset, especially as regards catfish farming.

Working in conjunction with two local catfish farmers in Nsukka, Enugu State, South-East Nigeria we set up three experimental project sites under the Lacuna Agriculture Fund Award 2020. We built IoT sensor units that were mounted on mobile tarpaulin fish ponds with spinach plant beds on top of them. Three ponds were set up in each of the farmer's sites, one set had the IoT units but no plant bed, while the other had neither IoT sensors nor plant beds, but was used as control. Meanwhile, in the University of Nigeria, Nsukka campus we set up 12 ponds, 9 of which were fitted with the IoT sensor units and plant beds, each comprising six sensors (temperature, pH, dissolved oxygen, turbidity, ammonia, and nitrate). The design and implementation of the IoT units and how they were used for data collection are described in the following sub-sections.

### Data collection

Data collection was done using an automated method with the use of IoT sensors to collect data on the water physicochemical properties which was done in real-time and transmitted to a cloud computing storage. Fish, plant, and algae growth, morphometry, and population dynamics were continuously assayed fortnightly. The detail of this approach is discussed in the next subsection.

#### Smart aquaponics dataset collection method

The experiment was conducted using six water quality sensors, out of which four were submerged in the pond water (temperature, turbidity, dissolved oxygen, and pH), while the other two were gas sensors, which were suspended over the water surface. These are ammonia and nitrate sensors. These sensors were calibrated according to industry specifications and programmed to collect water quality parameters in real-time and automatically upload the same to the cloud through the (IoT) [2] gateway. The process of calibration involved using some calibration solutions, in some cases (sodium hydroxyl), as in the cases of the pH and dissolved oxygen sensors. The sensors were connected to a 32-bit microcontroller known as ESP 32. The microcontroller has an in-built wireless (WiFi) module which enables the sensors' data to be automatically uploaded to a cloud computing platform, Thingspeak IoT cloud, through an IoT (edge) network. The C programming language was used to write the software program that controlled the sensors with the Arduino 1.8.4 integrated development environment (IDE) known as Sketch. The code was uploaded to the microcontroller. The sensor units were locally designed, constructed, and programmed. The IoT system was programmed to automatically read the six water quality parameters for each of the 12 aquaponics fish ponds and transmit them to the cloud storage hosted by Thingspeak (https://thingspeak.com/channels/1414062/) every 18 seconds, which is the minimum interval allowed by Thingspeak.

After testing the system for 10 months and comparing the values with ground truth values, we discovered that some sensors did not perform very well, notably the pH, and the two gas sensors. The pH sensor was not meant for such a volume of water as the fish pond. We discovered this later after more studies on the characteristics of the sensors. Meanwhile, the gas sensors (MQ-135 and MQ-137) for nitrate and ammonia, respectively, were reading high and unusual values.

**Fig. 1:**
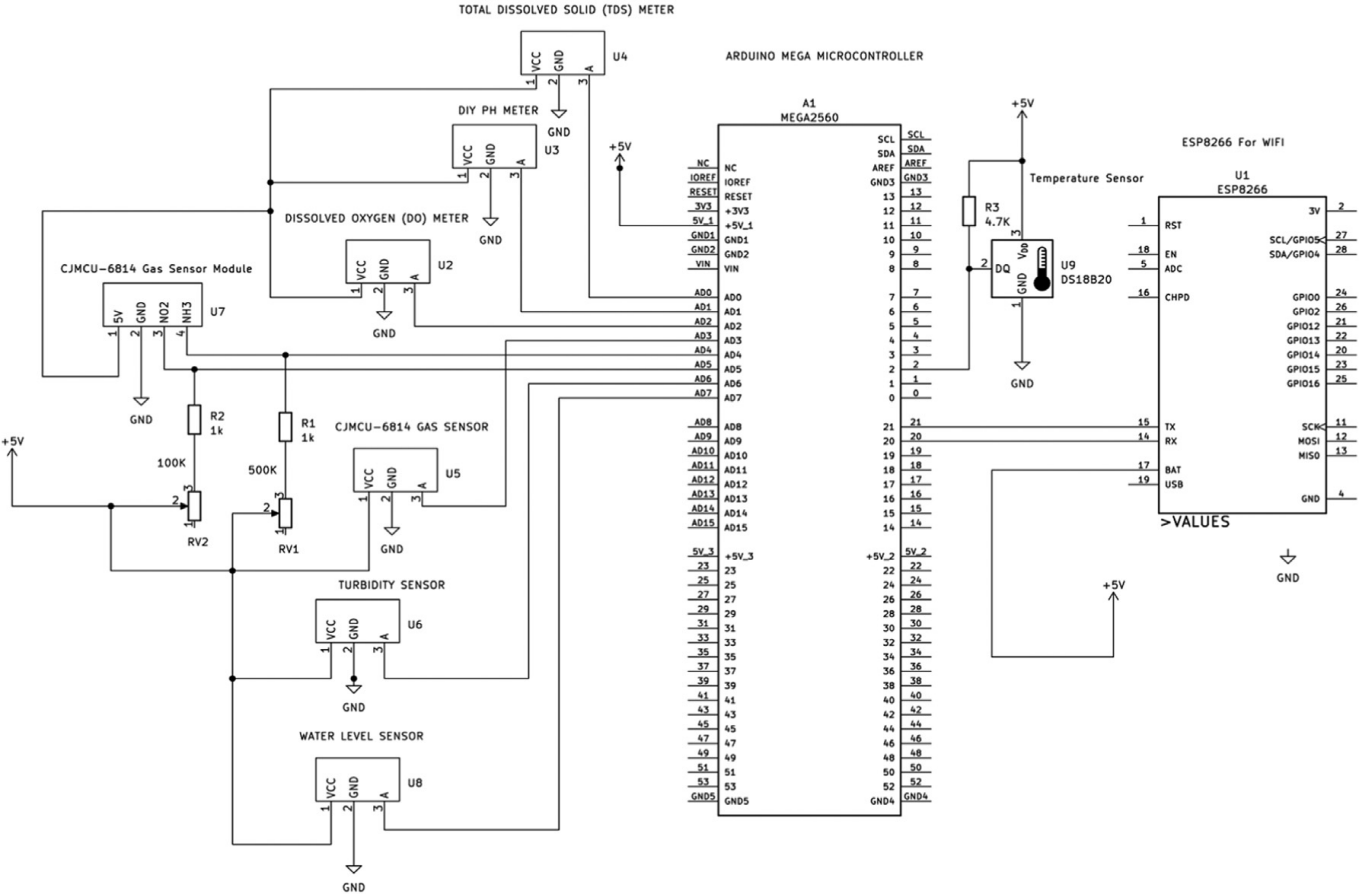
IoT circuitry schematic diagram of the improved system.

**Fig. 2:**
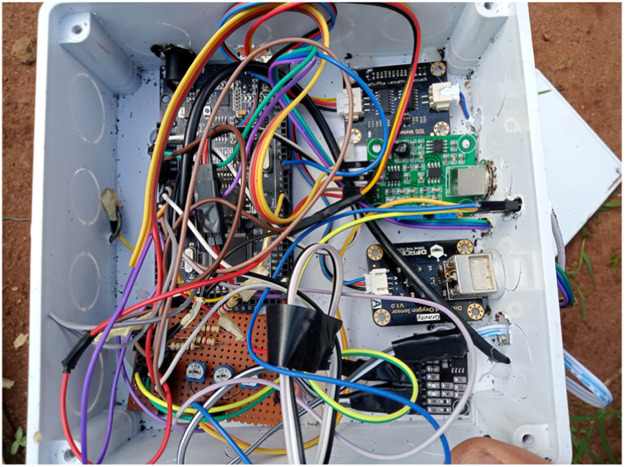
IoT Unit showing sensor circuitry.

**Fig. 3:**
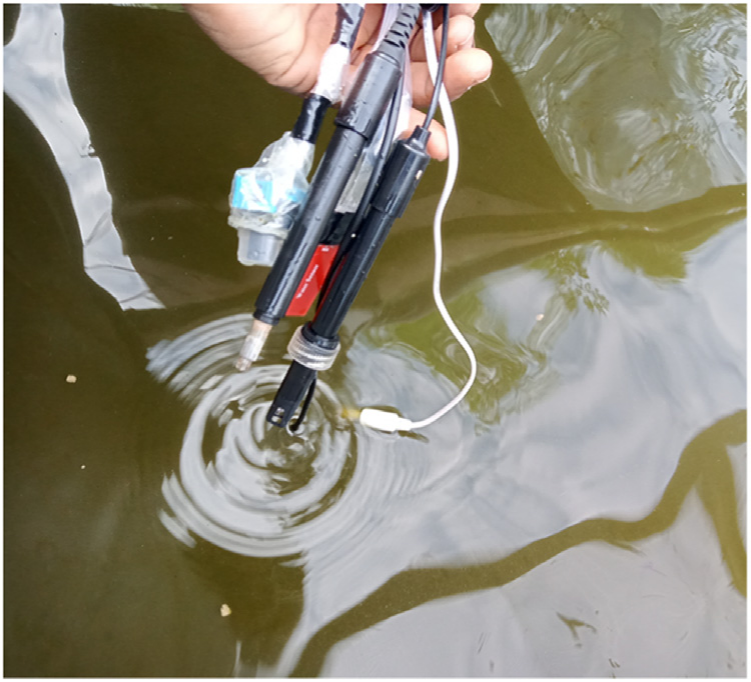
Aquaponics IoT submersed sensors.

We had to redesign the system using the Arduino Mega 2560 with built-in Wi-Fi as the microcontroller. We used a professional pH meter which can function large volume of water, and a new gas sensor for ammonia and nitrite, described in the next section. Fig 1 shows the circuit schematic diagram of the improved system while Fig. 2, Fig. 3 show the constructed new IoT circuitry and the sensors, respectively.

The core of the new system is the 32-bit Arduino 2560+WiFi R3, a new microcontroller (MCU) different from the original 2560 (see Fig. 1). This version has the Expressif's ESP8266 micro-WiFi module embedded in it. It has at its core the ATMega 2560, which is in the traditional Arduino mega. The MCU supports a voltage range from 5V to 12V, flash memory of up to 32MB, and CPU speed of up to 80MHz. It also comes with several [16,18] dual inline pins (DIP) switches and a table used for selecting the pins to connect different things depending on the objectives. The 2560 MCU also has 54 digital pins, whereas 15 pins support pulse width modulation (PWM), and 16 analog input pins and communicate via the 4x serial ports (UART).

Whereas the ESP8266 is primarily responsible for the connection to the Internet through a GSM router gateway to upload the datasets from the sensors to the cloud, the sensors are connected to the various I/O pins of the mega 2560 MCU. For instance, the DS18B20 submersible temperature sensor is connected to the digital pin 2, and the rest to analog pins as follows: TDS is connected to pin AD0, pH to AD1, dissolved oxygen to AD2, gas sensors; nitrate and ammonia to AD4 and AD5, respectively, while turbidity and water level sensors are connected to AD6 and AD7, respectively.

The MCU-MICS-6814, an air quality sensor that measures three gases; carbon monoxide, ammonia, and nitrite (CO, NH_3_, NO_2_) were installed to detect the ammonia and nitrite coming from the pond water. The MICS 6814 outdoor leak gas detector can detect the following gases: Carbon monoxide CO from 1 – 1000ppm; Nitrogen dioxide NO2: 0.05 – 10ppm; Ethanol C2H5OH: 10 – 500ppm; Hydrogen H2: 1 – 1000ppm; Ammonia NH3: 1 – 500ppm; Methane CH4 >1000ppm; Propane C3H8 >1000ppm; Iso-butane C4H10 >1000ppm.

Ammonia concentration in the fish pond can lead to mortality. Ammonia removal begins with converting it to nitrite by a good bacteria called Nitrosomonas. Nitrite is then converted to nitrate which is the final process of removing ammonia [17]. Nitrates are generally removed by plants, hence the need for the aquaponics system. Though nitrite and nitrate are not as harmful as ammonia but little concentration of them can lead to fish mortality.

#### Sensors specifications

In this section, we list the makes and models of the sensors used as well as the acceptable ranges for each of the water parameters.

(i) **DF Robot Gravity Analog pH Sensor Meter Pro Professional kit for Arduino Water Quality Surveillance Aquaculture*:*** This sensor is used to read the pH level of the pond water instead of the *DF Robot pH sensor probe for Arduino version 2.0* used in the initial IoT system. It monitors the water's pH and provides an early warning to avoid the water being acidic. Acceptable pH ranges for the catfish range from 6.5-9.0. pH also affects ammonia (NH_3_) concentrations. Each unit of change in pH is a factor of 10X of ammonia. Total ammonia and nitrogen, in addition to water temperature and pH, are needed to determine un-ionized ammonia (NH3) concentration**.** Total alkalinity = 50 -150 mg/L, the ability of the water to buffer changes in pH. pH of the aquaculture environment for the growth of fish and shrimp is about 6.5∼8.5.
(ii) ***DF Robot Dissolved Oxygen (DO) sensor probe for Arduino:*** DO has relatively lower solubility and availability in aquatic life than in terrestrial environments. Acceptable ranges for dissolved oxygen should be greater than 3mg/L, preferably 5mg/L, or more. The saturated dissolved oxygen in water with a water temperature of 25°C and a chlorinity of 0.0 is 8.26mg/L.
  ***DF Robot DC 5V TS-300B Turbidity Sensor Module***: Mixed Water Detection Module Water Quality Test Turbidity Transducer for Arduino.
(iii) ***Dallas DS18B20 temperature sensor.*** This sensor reads digital values. DSB18B20 has a Unique 1-Wire interface that requires only one port pin for communication. The ideal water temperature for catfish should not exceed 85°F (30 C).
(iv) ***Ammonia detection sensor NH_3_ gas sensor module MICS 6814:*** Un-ionized Ammonia (NH_3_) = Chronic or long-term problems 0.06 mg/L. Acute or short-term mortality 0.6 mg/L.
(v) ***Nitrite detection sensor NO_2_ gas sensor module MICS 6814:*** This sensor replaced the MQ 135 sensor**.** Ideally, nitrate levels in a freshwater aquarium should be kept below 20 mg/L. However, any changes should occur slowly, only removing less than 50 mg/L of the Nitrate per day.
(vi) ***Total Dissolved Solid (TDS):*** The TDS sensor was added to the new system which measures the amount of solluble solid in milligram that is dissolved in one liter of water (mg/l) or in parts per million (ppm). The more the solid dissolved in the water the less clean the water will be. The best values for fresh water fish pond should be less than 400ppm.

#### Machine learning model development

The datasets collected from both the old and new IoT units were taken from one of the 12 ponds, respectively and cleaned, preprocessed, and trained on the Google Colab platform using Python 3.7. Both datasets were downloaded from Kaggle where they were both stored separately. IoTPond_old, consists of 279,612 rows and 11 columns, while the dataset from the new sensor unit, IoTpond_new contains 128,206 rows and 11 columns. The purpose was to understand the correlation between the different dataset features and use machine learning models to predict fish growth in terms of length and weight based on various attributes of the water quality sensors.

## Method validation

**Fig. 4:**
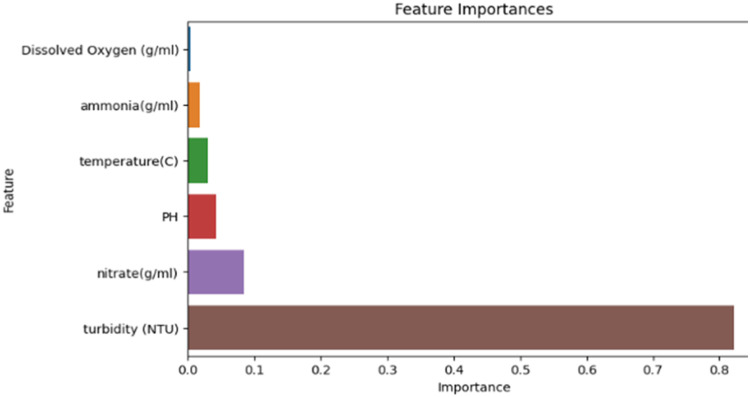
Machine learning result of first IoT the system.

**Fig. 5:**
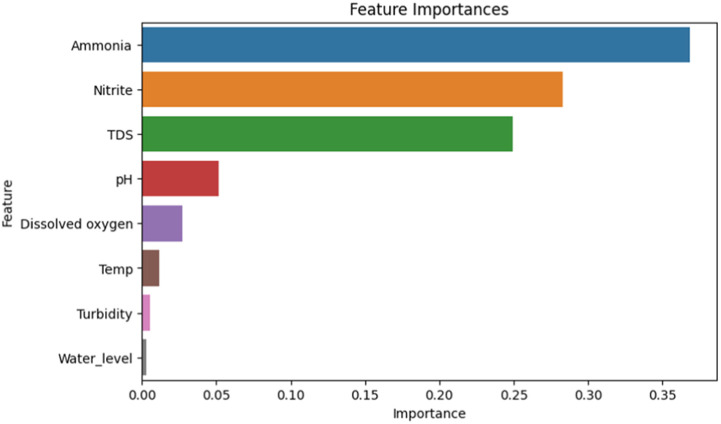
Machine learning result of the improved IoT system.

Fig. 4, Fig. 5 show the result of the correlation analysis of the water quality features in order of their importance from both the old and the new system, respectively. The new system shows a better result.

From Figs. 4 and 5, we notice that in the first IoT dataset, the most influential attributes for both systems were turbidity, nitrate, PH, and temperature. While for the new IoT dataset ammonia, nitrate, total dissolved solids (TDS), and pH are the most important attributes for determining the fish weight.

To predict fish length and weight, we utilized models like Linear Regression, Ridge Regression, Lasso Regression, K-Neighbors Regressor, and Decision Tree Regressor. We got their root mean squared error values (RMSE) for both fish weight and fish length and concluded from the models' RMSE that we can select Decision Tree Regressor for making our final predictions. The extreme gradient boost (XGBoost) Regressor model was used to enhance the performance of the models. It performed a randomized search with cross-validation to find the optimal hyperparameters for the models. The hyperparameters tuned were max_depth, learning_rate, n_estimators, gamma, and min_child_weight. The evaluation metric used for scoring was negative root mean squared error (RMSE).

After fitting the randomized search object to the training data, we obtained the best hyperparameters and evaluated the model on the test data. The test scores for fish length prediction for both versions of IoT sensor units are shown in Table 1.

**Table 1:** Fish length evaluation results for the two IoT systems.

Evaluation metrics	The result for the Old System	Result for New System
MSE	1.02725608411738	0.3770336613092
RMSE	1.01353642466237	0.61403066805271
MAE	0.54105020046009	0.27729398177683
R-Squared	0.99735129480191	0.99871034058614

The first IoT dataset and the new IoT dataset achieved test RMSE scores of 1.0128 and 0.6140, respectively for fish length prediction using Decision Tree Regressor. Further testing with the XGBoost Regressor achieved a test RMSE score of 7.0192 for fish weight prediction for the initial IoT dataset, and 0.7793 for the new IoT dataset. The new system consistently outperformed the old system in producing lower error values, and both tying on R-squared score, which is a measure of prediction accuracy.

The test scores for fish weight prediction for both versions of IoT sensor units are shown in Table 2.

**Table 2:** Fish weight evaluation results for the two IoT systems.

Evaluation metrics	The result for the Old System	Result for New System
MSE	49.26981352230902	0.51018846367
RMSE	7.019245936873064	0.71427478163
MAE	2.23104303871279	0.25627263382
R-Squared	0.999530930656313	0.99825094144

Just as discovered in Table 1, we also see that in Table 2 the improved system also outperformed the old system in all error values. Using the XGBoost Regressor on weight prediction led to the improved performance of the old IoT system, almost equaling the performance of the improved system.

**Fig. 6:**
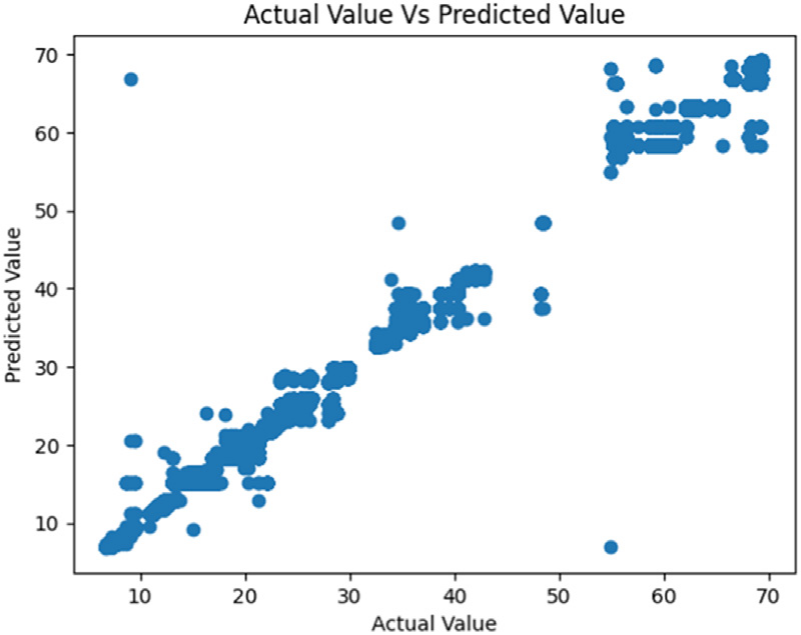
Fish weight prediction in the old IoT unit.

**Fig. 7:**
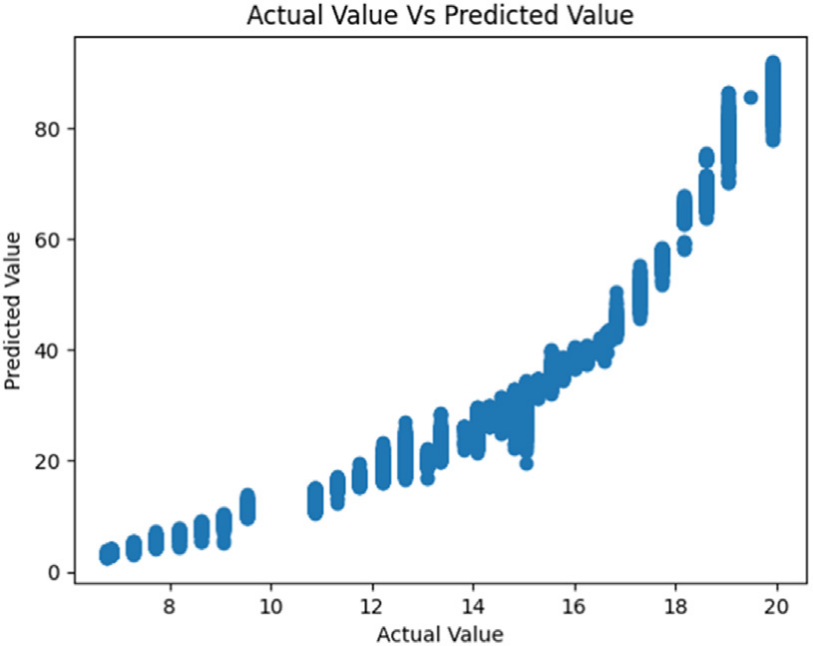
Fish weight prediction in the New IoT system.

Fig. 6, Fig. 7 show the regression plots for the predicted fish growth by weight against the actual weights in both the old and new IoT systems. Again, we see that the new system shows a better and more consistent smoother plot than the old system.

These evaluations clearly show that the new IoT system performed better than the initial one on all fronts. We also found out that XGBoost Regressor performed well for both fish weight and fish length models in both datasets and models. However, based on the models' RMSE, we recommend using Decision Tree Regressor for making final predictions for fish length and XGB Regressor for making final predictions for fish weight. We also discovered that the poor performance of the initial IoT system was due to poor-quality sensors and configuration of the sensors.

## Conclusion and future work

We were motivated by the need to assist farmers to find solutions to the stunted growth and mortality issues of their fishes. We also discovered that there was a water quality data and knowledge gap which made it difficult for farmers and researchers to effectively monitor and predict the performance of fishes in aquaculture ponds. Having received a grant from the Lacuna Agriculture Fund, we designed an IoT sensor system to monitor the physicochemical parameters of fish pond water. Working in conjunction with local farmers we collected the water parameters like temperature, dissolved oxygen, pH, turbidity, ammonia, and nitrate in real-time with the IoT sensors we designed and built. After several months of data collection, it was observed that some of the sensors did not deliver optimal values when compared with ground truths. This necessitated further research and the discovery of better-quality sensors for high quality datasets. A new system was designed and built with the new Arduino Mega 2560 + Wi-Fi microcontroller. Datasets from the old and new systems were both trained using different machine learning algorithms. The new IoT system performed much better than the initial one in all fronts showing that sensor quality, design experience, and research are major considerations in designing IoT systems.

The design and implementation of an improved IoT-Cloud orchestrated sensor system for aquaponics will provide a robust and scalable communication infrastructure, facilitating real-time monitoring, and data analytics in the aquaculture industry. This will also enhance the efficiency, productivity, and sustainability of aquaponics systems, contributing to the advancement of agriculture and food production. The cloud-based analytics platform will provide advanced insights, anomaly detection, and predictive maintenance, facilitating informed decision-making for system optimization.

Future work will focus on turning the best predictive machine learning model to a mobile application. Farmers will receive the app installed in their smart phones for monitoring water quality and predicting fish yield. Results gotten from the use of the app will be compared with baseline data for fish growth.

## Ethics statements

Our work was carried out in line with the ARRIVE guidelines  *and in accordance with the U.K. Animals (Scientific Procedures) Act, 1986 and associated guidelines;* *EU Directive 2010/63/EU for animal experiments* in terms of experimental design, sample size determination, and measurements. The fishes were randomly selected based on the sample size already determined per fish pond based on the stocking density. Measurements of weight and length were done and the fishes returned to the ponds.

## For a published article

C.N. Udanor, N.I. Ossai, B.O. Ogbuokiri, O.E. Nweke, P.O. Ugwoke, U.K. Ome (2021). A Pilot Implementation of a Remote IoT Sensors for Aquaponics System Datasets Acquisition, The Journal of Computer Science and it's Applications (JCSA), Vol. 28, Iss. 2. https://dx.doi.org/10.4314/jcsia.v28i2.1.

## Related research article

During the Lacuna Agriculture Fund 2020 project grant award [1] aimed at building agricultural datasets, our team designed and developed an IoT-sensors microcontroller-based system [[2], [19]] aimed at remotely monitoring and collecting physicochemical parameters from catfish aquaponics pond water. The primary deliverable from the project was a labeled dataset containing water quality parameters like water temperature, pH, dissolved oxygen, turbidity, dissolved ammonia, and nitrate from 12 aquaponics ponds. At the end of the project, the dataset was contributed to the Kaggle machine learning dataset at [3] under the Creative Commons Attribution 4.0 to the research community. This dataset comes in handy in developing machine learning models for predicting catfish health and yield.

In furtherance of the aforementioned research, the authors have been motivated to further improve the quality of the dataset to completely address the following problems: (i) the absence of accurate water quality datasets in any known machine learning data repository for the African Catfish (*Clarias gariepinus*), (ii) the high cost of generating a labeled African catfish dataset for machine learning without an expert, (iii) the challenge of time consumption and inaccurate generation of labeled data using manual methods, (iv) the need to recycle and reuse water given its scarcity in many African communities, (v) the need for an innovative ways of increasing productivity in a limited space offered by aquaponics, and (vi) the challenge of making aquaculture production widespread through the mobile and portable aquaponics model.

Given the above, the authors considered developing a scalable and robust GSM-Edge-Cloud aquaponics system with an efficient data collection mechanism from IoT sensors. The architecture of the proposed system includes an intelligent cloud-based analytics platform for real-time monitoring.

## CRediT authorship contribution statement

**A.H. Eneh:** Supervision. **C.N. Udanor:** Conceptualization, Methodology, Software, Visualization, Investigation. **N.I. Ossai:** Investigation. **S.O. Aneke:** Writing – original draft. **P.O. Ugwoke:** Writing – original draft, Validation, Data curation. **A.A. Obayi:** Writing – original draft. **C.H. Ugwuishiwu:** Writing – review & editing. **G.E. Okereke:** Software, Validation.

## Declaration of Competing Interest

The authors declare that they have no known competing financial interests or personal relationships that could have appeared to influence the work reported in this paper.

## Data Availability

Data will be made available on request.

## Supplementary material *and/or* additional information

Link to dataset:

Udanor collins, Blessing Ogbuokiri, Nweke Onyinye, Sensor Based Aquaponics Fish Pond Datasets, Kaggle, available at: https://www.kaggle.com/dsv/3748790, Kaggle, 2022.

## References

[1] Sensor based aquaponics fish pond datasets: IoT fish pond monitoring datasets, lacuna fund, Available at: https://lacunafund.org/datasets/agriculture/ (Accessed: 8th August, 2023).

[2] Udanor C.N., Ossai N.I., Nweke E.O., Ogbuokiri B.O., Eneh A.H., Ugwuishiwu C.H., Christiana A. (2022). An internet of things labelled dataset for aquaponics fish pond water quality monitoring system. Data in Brief.

[3] collins U., Ogbuokiri B., Onyinye N. (2022).

[4] Kiruthika S.U, Kanaga Suba Raja Dr.S., Jaichandran R. (2017). J. Adv. Res. Dyn. Control Syst..

[5] Chen C.H., Wu Y.C., Zhang J.X., Chen Y.H. (2022). IoT-based fish farm water quality monitoring system. Sensors.

[6] Danh L.V.Q., Dung D.V.M., Danh T.H., Ngon N.C. (2022). Design and deployment of an IoT-based water quality monitoring system for aquaculture in Mekong Delta. Int. J. Mech. Eng. Robot. Res..

[7] Nocheski S. and Naumoski A. Scientific technical union of mechanical engineering “Industry 4.0” Issue, 2018.

[8] Saha S., Rajib R.H., Kabir S. (2018). International Conference on Innovations in Science, Engineering and Technology (ICISET).

[9] Kim Y., Lee N., Kim B., Shin K. (2018). International Symposium on Computer, Consumer and Control (IS3C). 06-08 December.

[10] Tamim A.T., Begum H., Shachcho S.A., Khan M.M., Yeboah-Akowuah B., Masud M., Al-Amri J.F. (2022). Development of IoT based fish monitoring system for aquaculture. Intell. Autom. Soft Comput..

[11] Gao G., Xiao K., Chen M. (2019). An intelligent IoT-based control and traceability system to forecast and maintain water quality in freshwater fish farms. Comput. Electron. Agricult..

[12] Ramya A., Rohini R., Ravi S. (2019). IoT based smart monitoring system for fish farming. Int. J. Eng. Adv. Technol..

[13] Yang X., Cao D., Chen J., Xiao Z., Daowd A. (2020). AI and IoT-based collaborative business ecosystem: a case in Chinese fish farming industry. Int. J. Technol. Manag..

[14] Agossou B.E, Toshiro T. (2021). GoodIT '21: Proceedings of the Conference on Information Technology for Social Good September.

[15] Ahmed M., Rahaman Md.O, Rahman M., Kashem M.A. (2019). 2019 IEEE International Conference on Sustainable Technologies for Industry 4.0 (STI)**,** 24-25 December.

[16] Louis, L.&. Kumar, A. Implementation of closed loop based scan mechanism, 2021, 309-313. doi:10.1109/CCIntelS.2015.7437930.

[17] Eco-filtration. How to cure and prevent dangerously high nitrate and nitrite levels in a fish pond. Available at: https://www.eco-filtration.co.uk/prevention-and-treatment-for-high-nitrate-nitrite-levels/#:∼:text=Nitrates%20are%20a%20natural%20by-product%20of%20the%20bacterial,to%20fish%20mortality%20if%20not%20kept%20in%20check, (Nov 30, 2021).

[18] Koyanagi F., AutoDesk Instructables, Arduino MEGA 2560 With WiFi Built-in - ESP8266. Available at: https://www.instructables.com/Arduino-MEGA-2560-With-WiFi-Built-in-ESP8266/(Retrieved: 25th June, 2023).

[19] Udanor C.N., Ossai N.I., Ogbuokiri B.O., Nweke O.E., Ugwoke P.O., Ome U.K. (2021). A pilot implementation of a remote IoT sensors for aquaponics system datasets acquisition. J. Comput. Sci. Appl. (JCSA).

